# Exploring Nurses’ and Nursing Students’ Attitudes Toward Coercive and Technological Measures in Mental Health: A Conceptual Framework and Study Protocol

**DOI:** 10.3390/nursrep14040301

**Published:** 2024-12-20

**Authors:** Giuliano Anastasi, Roberto Latina, Yari Longobucco, Alessandro Stievano, Stefano Bambi

**Affiliations:** 1 Department of Biomedicine and Prevention, University of Rome Tor Vergata, 00133 Rome, Italy; 2 Department of Health Promotion, Mother and Childcare, Internal Medicine and Medical Specialties, University of Palermo, 90127 Palermo, Italy; roberto.latina@unipa.it; 3 Department of Health Sciences, University of Florence, 50139 Florence, Italy; yari.longobucco@unifi.it (Y.L.); stefano.bambi@unifi.it (S.B.); 4 Department of Clinical and Experimental Medicine, University of Messina, 98125 Messina, Italy; alessandro.stievano@unime.it

**Keywords:** nursing, mental health, psychiatry, security measures, coercion, technologies, safety, attitudes

## Abstract

Background/Objectives: The use of coercive measures (CMs) and security technologies (STs) in mental healthcare continues to raise ethical and practical concerns, affecting both patient and staff well-being. Mental health nurses (MHNs) and nursing students (NSs) play a key role in the decision-making process regarding these interventions. However, their attitudes, particularly toward STs, remain underexplored in Italy. This study protocol aims to introduce a new conceptual framework and investigate Italian MHNs’ and NSs’ attitudes toward CMs and STs in mental health settings. Additionally, it will explore the influence of sociodemographic and psychological factors, including stress, anxiety, depression, stigma, and humanization on these attitudes. Methods: The research will be conducted in two phases. Phase 1 involves a national survey of a convenience sample of MHNs and NSs to assess their attitudes and related factors. Phase 2 includes qualitative interviews with a purposive sample of MHNs and NSs to explore participants’ perspectives on STs in more depth. Quantitative data will be analyzed using descriptive and inferential statistics, while qualitative data will be examined through thematic analysis. Conclusions: This study protocol seeks to enhance our understanding of MHNs’ and NSs’ attitudes toward the use of CMs and STs in mental health settings, identifying key factors influencing these attitudes. The findings aim to inform policy development, education programs, and clinical practices in both the Italian and international panoramas. Additionally, the proposed conceptual framework could guide future research in this field.

## 1. Introduction

The primary aim of psychiatric care is ‘to keep patients and others safe’ [1], highlighting the value of safety in mental health [2]. However, mental health settings face several challenges that can compromise safety [3]. These challenges include patient falls [4], adverse reactions to therapeutic interventions [5], and difficult patient behaviors, such as absconding [6]. Violence remains a critical issue, with a significant number of psychiatric inpatients exhibiting violent behavior [7], engaging in self-harm, or revealing suicidal tendencies [8]. Furthermore, up to 85% of mental health staff report experiencing assaults [9], which can lead to physical injuries [10] and psychological consequences [11].

### 1.1. Coercive Measures

These safety challenges are potential antecedents of coercion [12], precipitating the use of coercive measures (CMs) [13,14]. CMs are defined as ‘a medical measure carried out against the patient’s self-determined wishes or in spite of opposition’ [15]. Formal CMs include involuntary admission, observation, seclusion, forced medication, and restraint [12], while informal CMs encompass practices such as persuasion, manipulation, and threat [16].

Despite their common use, CMs raise significant concerns [12]. Patients subject to CMs may experience fear, anger, trauma [17,18], physical injury, or even death [19]. Staff implementing CMs can face the risk of physical harm [20], frustration, guilt, and ethical dilemmas [21,22,23]. Additionally, CMs can damage therapeutic relationships, infringe on patients’ rights, and reinforce stigma [12,24]. Although policies such as the Safewards Model [25] have shown promise to reduce CMs, they remain prevalent in contemporary mental health practices [26], and mental health staff still consider CMs necessary for maintaining safety [27,28].

### 1.2. Security Technologies

In light of the challenges and ethical concerns surrounding CMs, there is growing interest in alternative strategies to enhance safety in mental healthcare [29,30]. One emerging solution is the use of security technologies (STs), such as surveillance cameras, body-worn cameras, personal or fixed alarms, and metal detectors [31,32,33,34]. These technologies can enhance safety by enabling remote patient monitoring [31], facilitating rapid incident response [35], and preventing the introduction of dangerous items [32]. International guidelines support the use of STs such as surveillance cameras and alarms to mitigate suicide and violence in mental health [36,37], while in Italy, psychiatric forensic facilities already integrate them [38] following national policies [39,40].

However, research on the use of STs in mental health settings is still emerging [31,33,34]. While some studies highlight their values in enhancing safety and well-being [32,41], others identify potential risks, including increased property damage and violence [42,43]. Ethical concerns related to patient consent, privacy, and dignity also complicate their adoption [31,44,45].

### 1.3. The Role of Mental Health Nurses and Nursing Students

Mental health nurses (MHNs) are crucial in ensuring safety in psychiatry, also using CMs [46]. Therefore, extensive research has reviewed MHNs’ attitudes toward CMs [21,23,29], highlighting mixed findings and the need for further studies [23,27,29]. For instance, MHNs’ attitudes toward CMs are underexplored in Italy [47], with studies predominantly focused on general attitudes toward mental illness [48] and informal coercion [49]. Similarly, while international research is beginning to explore MHNs’ perspectives on certain STs such as body cameras [33,44,45], exploration of the attitudes towards an extensive range of STs is still lacking.

Nursing students (NSs) also participate in psychiatric care during their internship [50,51]. They actively witness, assist, and implement CMs [52,53], and can develop favorable attitudes toward them [53,54,55]. Furthermore, their perceptions could strongly predict the application of CMs [56]. However, despite their involvement, NSs’ attitudes toward both CMs and STs are underexplored.

### 1.4. Conceptual Framework

The conceptual framework guiding this research integrates Johansen’s [57] and Moylan’s [58] decision-making models, enriched by key theoretical insights from the literature. Johansen’s model highlights how education, experience, values, knowledge, and stress influence decision-making in nursing practice [57]. Moylan’s model focuses on how MHNs navigate among perceived risks, available information, and personal values when selecting CMs in response to patient aggression [58]. Both models are pertinent to MHNs, who are described as ‘leaders during the application of restrictive practices in psychiatry’ [59], with substantial responsibility for initiating CMs [27].

This framework is further supported by the Theory of Planned Behaviour [60] and the Normative-Affective Decision-Making Theory [61], which highlights how beliefs, social norms, and emotional factors shape attitudes and behaviors. This aligns with existing research suggesting that both personal and normative factors have a strong influence on attitudes toward the use of CMs and STs [23,62,63].

**Figure 1:**
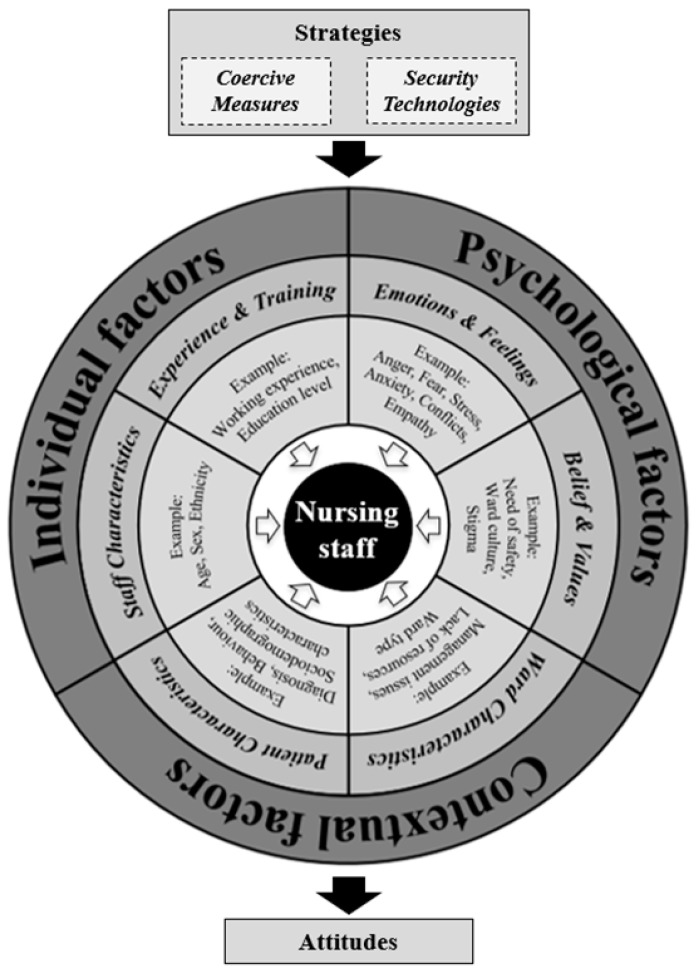
The conceptual framework with the factors involved in shaping the attitudes toward coercive measures and security technologies in mental health nursing.

Based on these models and the existing literature, the proposed framework (see Figure 1) suggests that MHNs’ and NSs’ attitudes toward CMs and STs are shaped by three main categories of factors: individual, psychological, and contextual factors.

#### 1.4.1. Individual Factors

Staff characteristics: Evidence suggests that sociodemographic characteristics may influence attitudes and behaviors toward the use of CMs. Male MHNs are more likely to use CMs compared to females [13,14,64,65]. Shifts involving male MHNs have been associated with increased use of CMs [63]. Male NSs also express greater confidence in applying CMs [54,66]. However, some research suggests that female MHNs may exhibit more favorable attitudes toward CMs [65], with their presence linked to higher rates of seclusion [67]. Age is another important factor: younger MHNs tend to be more supportive of CMs [68], while older MHNs often express ethical concerns [69]. Additionally, MHNs’ ethnicity has been identified as influencing CMs’ use [70].

Experience and Training: The relationship between experience, training, and attitudes toward CMs is complex, with research providing mixed results. Some studies indicate that higher education correlates with increased CMs use [63,64], while others show that more educated MHNs are less supportive [14,67]. Work experience also plays a significant role. More experienced MHNs often resort to CMs less frequently [67], and hold critical views on their use [22,66]. In contrast, some studies report that experienced MHNs continue using CMs consistently [68,71,72]. Less experienced MHNs may view CMs as beneficial interventions [73], and adopt more restrictive approaches [65,67,73]. Additionally, feelings of being underskilled [74] or inexperienced in mental health nursing [64,75] are associated with higher reliance on CMs.

#### 1.4.2. Psychological Factors

Emotions and Feelings: Emotional states can influence decisions to use CMs in psychiatric settings. Emotions such as anger, fear, stress, and anxiety can increase the likelihood of MHNs resorting to CMs [28,46,68]. Stress and anxiety can undermine MHNs’ confidence in managing aggression, increasing their reliance on CMs [22]. NSs show similar tendencies, supporting the use of CMs when experiencing anxiety and fear [76]. Additionally, team dynamics and interpersonal conflicts among nursing staff are linked to higher CM use [25,77]. Conversely, positive emotional traits such as empathy are associated with a reduced likelihood of employing CMs [78].

Beliefs and Values: Beliefs and values can shape MHNs’ attitudes toward CMs. A greater perception of risk is typically associated with increased use of CMs [29,79]. Similar trends are observed among NSs [76]. Ward culture and norms further influence the acceptance or rejection of CMs [72,73,75,80], leading to variations in CM use across units and countries [13,64]. Familiarity with CMs also predicts more favorable attitudes among MHNs and NSs [54,55,66,76,81]. Additionally, stigma toward mental illness, common among MHNs and NSs [82,83], can influence the approval of CMs [84].

#### 1.4.3. Contextual Factors

Ward characteristics: The wards’ physical environment and operational structure can influence the use of CMs [80]. Working in open versus closed units, or acute versus chronic settings, shapes MHNs’ attitudes toward CMs [64,65,71,77]. Heavy workloads and staffing shortages increase the likelihood of using CMs, as MHNs may lack sufficient resources to manage incidents effectively [79,85]. For instance, shifts with less experienced staff are linked to higher CMs use [64]. Additionally, poor leadership, inadequate safety protocols, and organizational dysfunction contribute to increased reliance on CMs [74,77].

Patient characteristics: Patient-specific factors, such as diagnosis severity and type, are key determinants in decisions to implement CMs [13,85,86]. Patients exhibiting aggressive behaviors or refusing treatment are more likely to experience CMs [14,62,63,65]. Sociodemographic factors also play a role: patient sex [13,71,86,87], age [13], ethnicity [13,87], and marital status [87] can influence CMs use. Additionally, patients admitted involuntarily are more likely to be subjected to CMs compared to voluntary admissions [13].

## 2. Materials and Methods

### 2.1. Research Hypothesis

Based on the literature and the conceptual framework developed, the following research hypotheses were formulated:

(1) Attitudes toward coercive measures and security technologies in psychiatric settings differ between mental health nurses and nursing students.
(2) In mental health nurses and nursing students, attitudes toward coercive measures and security technologies in psychiatric settings vary depending on the specific type of strategy considered.
(3) In mental health nurses and nursing students, attitudes toward coercive measures and security technologies in psychiatric settings are influenced by sociodemographic and psychological variables, including levels of depression, anxiety, stress, stigma toward mental illness, and humanization of care.

### 2.2. Objectives

The primary aims of the research are as follows:

- Assess the attitudes of mental health nurses and nursing students toward coercive measures and security technologies in Italian psychiatric settings.
- Investigate the associations between these attitudes and sociodemographic and psychological variables in mental health nurses and nursing students.

The secondary aim is as follows:

- Qualitatively explore the perspectives of mental health nurses and nursing students regarding the appropriateness of security technologies in mental health settings.

### 2.3. Project Design and Phases

This project adopts a non-experimental, mixed-methods, multicenter design, with an estimated duration of 12 months. It consists of two interdependent phases:

- Phase 1—Cross-sectional National Survey: This phase addresses the primary objectives and serves as the foundation for the protocol. A national survey will provide a comprehensive ‘snapshot of how things are at a specific time’ [88]. This approach effectively identifies trends and variations across populations, addressing gaps in existing research [89,90]. The survey will adhere to the STROBE (Strengthening the Reporting of Observational studies in Epidemiology) guidelines to ensure rigor [91].
- Phase 2—Qualitative Descriptive Study: Building on the survey findings, this phase focuses on the secondary aim. The rationale for conducting a qualitative descriptive study is to ‘provide straightforward descriptions of experience and perceptions’ [92]. This approach is recommended in mixed-methods research to further explore quantitative results [92]. The study will adhere to the COREQ (Consolidated Criteria for Reporting Qualitative Research) guidelines to ensure quality and transparency [93].

### 2.4. Study Population

In Italy, nursing education consists of a three-year university program leading to a bachelor’s degree in Nursing, designed to provide general nursing competencies [94]. This program can offer exposure to mental health knowledge through multidisciplinary courses and internships within National Health Service institutions [95]. However, research indicates that national nursing curricula show considerable variability and lack standardization [96,97], and Italian nurses often graduate with limited psychiatric nursing education [47]. Furthermore, while post-graduate courses in mental health nursing are available, they are not mandatory for employment in psychiatric settings [98]. Based on this context, the study population is defined as follows:

- Nursing students: Students enrolled in a general nursing program, regardless of year of study, theoretical training, or mental health internship experience.
- Mental health nurses: Nurses employed in mental health settings, regardless of specialized training in mental health nursing.

### 2.5. Phase 1: Cross-Sectional National Survey

#### 2.5.1. Sample and Sampling Procedure

Given the impracticality and costs of collecting data from the entire target population [89], this study will use convenience and snowball sampling methods [89,99]. These methods ensure efficient recruitment while aiming to achieve a sample representative of the population of interest [89,99]. Participants will be selected based on the following inclusion criteria: (1) consent to participate; (2) proficiency in Italian; (3) students enrolled in a general nursing program; (4) nurses employed in a mental health setting. To ensure consistency and relevance in the responses, the following exclusion criteria will be applied: (1) students who have suspended their program for more than one year; (2) nurses who have suspended work for more than one year; (3) nurses with less than one year of work experience in mental health.

#### 2.5.2. Recruitment Process

Recruitment will initially focus on MHNs employed at the University Hospital and NSs enrolled in the School of Nursing at the primary study center. Recruitment invitations will be distributed via institutional email and local announcements [99]. To ensure national representation, recruitment will extend beyond the primary center. Nursing directors and chief nurses from mental health departments and units across Italian hospitals will be contacted to facilitate survey dissemination among MHNs. Similarly, nursing professors at Italian universities will be contacted to assist in enrolling NSs. Additionally, to align with modern recruitment practices, social media platforms, national mental health nursing associations, and nursing forums will be used to disseminate the survey, engaging MHNs and NSs outside academic or institutional networks.

All potential participants will receive a cover letter explaining the study’s purpose, procedures, and ethical considerations, along with a link to the online questionnaire. Participation will require completing an informed consent form before accessing the survey, ensuring transparency, voluntary participation, and adherence to ethical standards.

#### 2.5.3. Sample Size

According to the Italian Ministry of Health, there are 14,904 MHNs employed in public and private mental health settings across Italy [100]. Additionally, data from the Italian Ministry of University and Research indicate that 55,842 NSs are enrolled in nursing programs nationwide [101,102,103]. Given the lack of prior studies assessing attitudes toward CMs and STs among Italian MHNs and NSs, the prevalence of positive or negative attitudes in these populations is unknown. As a result, the prevalence will be assumed at 50%, which, according to the World Health Organization guidelines [104] and established methodologies for sample size determination in survey research [105], leads to the maximum sample size required.

The required sample size for each population will be determined using Cochran’s formula as outlined by Barlett and colleagues [105]:

$$n=\frac{t^{2}*[p*\left(1-p\right)]}{e^{2}}$$

where n = required sample size; t = t-value (1.96 for a 95% confidence interval in populations with more than 120 subjects); p = assumed proportion (0.5 for a prevalence of 50%); and e = acceptable margin of error. This formula has been used in other recent healthcare publications [106].

Using this approach, considering a 95% confidence interval, a 5% margin of error, and a 50% assumed prevalence for maximization, the sample size required is shown below:

$$n=\frac{1.96^{2}*[0.5*\left(1-0.5\right)]}{0.05^{2}}\approx 384$$

Thus, to ensure the statistical significance of the results, the survey will include at least 384 participants from each population.

#### 2.5.4. Data Collection and Questionnaire

Data will be collected using an online self-report questionnaire hosted on Google Forms ©, designed according to best practices in survey methodology [89]. Online surveys are particularly suitable for research on sensitive topics, as they promote honest responses, reduce social desirability bias, and enhance data reliability [107].

The questionnaire consists of seven sections with a total of 90 closed-ended items. The first section gathers sociodemographic information, the second collects professional or academic data (distinguishing between MHNs and NSs), and the third evaluates attitudes toward CMs and STs. The subsequent sections include three validated Italian instruments to measure psychological variables, and the final section assesses participants’ willingness to engage in the qualitative study.

Due to the lack of existing instruments to evaluate MHNs’ and NSs’ attitudes towards CMs and STs, the third section was developed by the authors. The method employed mirrors the approach used by Bowers and colleagues [108] in designing a survey to explore safety procedures and measures used in psychiatric units. The development process involved establishing the theoretical framework, performing a literature review, and conducting a focus group involving the authors and five experts: two mental health chief nurses, two nursing professors, and a statistician. To ensure clarity and relevance, content and face validity testing was conducted using established methodologies [109,110]. The content validity ratio (CVR) ranged from 0.60 to 1, exceeding the minimum threshold of 0.49 [111]. The item-level content validity index (I-CVI) ranged from 0.80 to 1, surpassing the cut-off of 0.70 [112]. Additionally, the scale-level content validity index (S-CVI/Ave) was 0.90, above the recommended threshold of 0.80 [109]. Evaluation of face validity further confirmed that the questions were clear, relevant, and applicable to the study’s objectives. Consequently, the items were deemed valid for administration.

The entire questionnaire was then tested on ten participants from each population to evaluate completion times, which ranged from 16 to 30 min. This length aligns with recommendations for maintaining participant engagement in online surveys [113]. The completion times are also consistent with the literature on web survey responses, where participants typically spend between one and ten seconds per question [114].

The questionnaire will be structured as follows:

1. Sociodemographic variables: In this six-item section, participants will provide demographic information including sex, age, marital status, ethnicity, and spiritual beliefs. They will also indicate their current role (MHN or NS) to ensure they receive the appropriate subsequent questions. The anticipated completion time is two minutes.
2a. Questions for MHNs: In this 14-item section, MHNs only will answer questions about their working role (i.e., clinical or chief nurse), educational level (e.g., bachelor’s or master’s degree), education in mental health nursing (i.e., yes or no), years of work experience, years of work experience in mental health, geographical region, work setting (i.e., acute, community, residential), contract type (i.e., permanent or fixed-term), work schedule (i.e., full-time or part-time), and work pattern (i.e., shift or daytime). They will also assess their perceived workplace safety, job satisfaction, intention to change work settings, and inclination to leave the profession, using a 5-point Likert scale (1 = not at all; 5 = very). The anticipated completion time is five minutes.
2b. Questions for NSs: In this nine-item section, NSs only will be asked about the geographical region where their program is located, academic status (i.e., full-time or part-time student), year of enrolment, education in mental health nursing (i.e., yes or no), internship pattern (i.e., shift or daytime), and hours spent in mental health internships. Additionally, they will evaluate their academic satisfaction, intention to leave the program, and interest in working in the mental health field using a 5-point Likert scale (1 = not at all; 5 = very). The anticipated completion time is four minutes.
3. Attitudes towards CMs and STs: In this eight-item section, participants will assess the appropriateness of CMs and STs commonly used in psychiatry according to the literature, including physical restraints, pharmacological restraints, locked ward doors, video surveillance systems, body-worn cameras, fixed alarms, portable alarms, and metal detectors. Responses will be collected using a 5-point Likert scale (1 = not at all appropriate; 5 = very appropriate). The anticipated completion time is four minutes.
4. Depression Anxiety Stress Scale (DASS-21): This 21-item scale measures levels of depression, anxiety, and stress [115]. Participants will report the frequency of symptoms experienced over the past week on a 4-point Likert scale (0 = never; 3 = almost always). The total score for each dimension will be calculated. Internal consistency values are α = 0.82 for depression; α = 0.74 for anxiety; α = 0.85 for stress. The anticipated completion time is seven minutes.
5. Opening Minds Stigma Scale for Healthcare Providers (OMS–HC): This 12-item scale measures stigma towards mental illnesses, focusing on attitudes towards people with mental illnesses and disclosure of mental illness [116]. Participants will indicate their agreement with each statement using a 5-point Likert scale (0 = strongly disagree; 4 = strongly agree). Higher scores indicate greater levels of stigma. Internal consistency values are α = 0.74 for stigma towards people with mental illness and α = 0.86 for stigma towards disclosure. The anticipated completion time is five minutes.
6. Healthcare Professional Humanization Scale (HUMAS-I): This 19-item scale evaluates the humanization of care across five dimensions: affection, self-efficacy, emotional understanding, optimistic disposition, and sociability [117]. Participants will rate the frequency of their experiences using a 5-point Likert scale (1 = never; 5 = always). Aggregate scores will be calculated for each dimension and the overall scale. Internal consistency values are as follows: α = 0.87 for affection; α = 0.82 for self-efficacy; α = 0.81 for emotional understanding; α = 0.82 for optimistic disposition; α = 0.70 for sociability. The anticipated completion time is seven minutes.
7. Willingness to participate in an interview: A dichotomous question (yes/no) will determine participants’ willingness to engage in the interview.

#### 2.5.5. Data Analysis

Descriptive and bivariate statistical methods will be used to analyze the data. Frequencies, percentages, medians, means (Ms), standard deviations (SDs), and quartiles will be calculated for all variables. To verify the assumptions required for parametric testing, normality will be assessed using the Shapiro–Wilk test, and homogeneity of variances will be evaluated with Levene’s test [118]. Correlations will be investigated through Pearson’s correlation for normally distributed continuous data, while Spearman’s rho will be used for non-normally distributed continuous data, ordinal data, or data with relevant outliers [119]. Differences in attitudes between groups will be assessed using independent samples t-tests for two-group comparisons and one-way ANOVA for comparisons involving more than two groups [120]. Significant findings from ANOVA will be further analyzed with Tukey post hoc tests to identify group differences. Potential confounding variables, such as sociodemographic and psychological factors, will be controlled through stratification, ensuring that these variables remain constant within subgroups [121]. Missing data will be excluded from the dataset to mitigate the risk of bias. All statistical analyses will be performed using SPSS© software (version 26), with a significance level set at *p* < 0.05.

### 2.6. Phase 2: Qualitative Descriptive Study

#### 2.6.1. Sample and Sampling Procedure

For the qualitative phase of the project, a purposive sampling strategy will be employed. This strategy, with a focus on maximum variation, is recommended for qualitative research as it ensures a heterogeneous sample, allowing information-rich cases to be selected [122,123]. This approach is needed to capture a wide range of perspectives and experiences related to the research topic [92]. Participants will have the option to indicate their willingness to join the qualitative phase (Phase 2) by responding to the final question of the questionnaire administered in Phase 1. Sampling will thus be performed on the MHNs and NSs who expressed their willingness to participate in the interview.

#### 2.6.2. Recruitment Process

The recruitment strategy will ensure comprehensive representation across key demographic variables, such as sex, age, geographical location, and educational background, enhancing the richness and diversity of the data. Recruitment will continue until data saturation is achieved, defined as the point at which no new information or themes emerge from the data [124]. Data saturation is considered the gold standard in qualitative research [122,125,126], ensuring the thoroughness and reliability of the findings [125,127]. Participants will be contacted via the email address provided in Phase 1. They will receive an invitation that includes a cover letter with detailed study information, informed consent forms, and options for scheduling the interview.

#### 2.6.3. Sample Size

In the literature, data saturation in qualitative research typically occurs between nine and 17 interviews [126,128], with saturation often reached around the 12th interview [125,128]. Accordingly, the initial target is to conduct interviews with 15 MHNs and 15 NSs. However, since qualitative research is inherently iterative, involving concurrent sampling, data collection, and analysis [128], recruitment and interviews will continue if initial rounds do not achieve saturation.

#### 2.6.4. Data Collection and Interview

Qualitative data will be collected through semi-structured interviews, each lasting approximately 60 min. To ensure participant comfort and enhance data quality, interviews will be conducted in the format preferred by the participant, whether in-person, by telephone, or online via videoconferencing software (Google Meets ©, version 280.0). Providing this flexibility helps create a setting that encourages open and genuine disclosure [129].

The interviews will use open-ended questions derived from the literature, designed to explore participants’ perceptions in depth and generate richer data [130]. Open-ended questions allow participants to express their insights freely, ensuring the data reflect the complexity of their experiences. All interviews will be audio-recorded with participants’ consent to enable precise transcription and ensure the data are captured verbatim [131]. Additionally, researchers will take detailed notes during interviews to document non-verbal cues and contextual details that may not be captured in the audio recordings [124].

The interview will be guided by five prompting questions (see Table 1), designed to generate detailed responses on both practical and ethical considerations regarding the use of STs in psychiatric settings.

#### 2.6.5. Data Analysis

Data analysis will be conducted using NVivo^®^ software (version 14) and will adopt an inductive approach following the classic method of content analysis [92,123]. The dataset will include interview transcriptions and detailed notes taken during the interviews. These transcriptions will be digitally processed to facilitate the coding, identification of categories, and abstraction into themes. The analysis will involve two coding cycles: preliminary coding and targeted coding [132]. In the first cycle, open coding will be used to assign board labels to data segments, briefly describing their content. During the second cycle, these preliminary codes will be refined and synthesized into specific themes that accurately reflect participants’ experiences and perspectives.

#### 2.6.6. Rigor

To ensure rigor and trustworthiness, the criteria established by Lincoln and Guba [124] will be followed. These criteria are widely recognized as the gold standard for ensuring the reliability and validity of qualitative research [92,127]. The following strategies will be employed to uphold these criteria:

- Bracketing (Suspension of Judgment): Researchers will set aside any preconceptions or biases during data collection and analysis to ensure objectivity and minimize potential bias.
- Coding Manual (Codebook): A structured codebook will be developed to guide the coding process, ensuring consistency, transparency, and coherence in interpretation.
- COREQ Checklist: The Consolidated Criteria for Reporting Qualitative Research (COREQ) checklist [93] will be used to ensure comprehensive and transparent documentation of the study’s procedures and findings.

To further enhance the validity of the findings, the identified themes will be presented to participants in follow-up interviews. This process allows participants to confirm the accuracy of the researcher’s interpretations or suggest adjustments, thereby strengthening the credibility and validity of the results.

### 2.7. Ethical Considerations

The research protocol has been reviewed and approved by the Local Ethics Committee of Palermo 1 (protocol number 204/2024, dated 28 March 2024). The project adheres to international ethical standards, including the Declaration of Helsinki [133], Good Clinical Practice (Ministerial Decree 15/07/1997 and subsequent amendments), and the Oviedo Convention. It also complies with Italian laws about non-interventional studies, the General Data Protection Regulation (GDPR) 2016/679, and the Italian Personal Data Protection Code (Legislative Decree 101/2018).

Participants will receive comprehensive information about the study, including its purpose, procedures, the voluntary nature of participation, and the right to withdraw at any time without consequences. They will be informed about confidentiality, anonymity, and data management. Informed consent will be required according to the Italian Legislative Decree n. 196 of 30 June 2003. Signed consent forms and collected data will be securely stored on a password-protected hard disk drive, accessible only to the principal investigators. Personal information, such as email addresses, will be used only for communication purposes and excluded from data analysis. Data will be presented in an aggregate form, and any personal identifiers will be replaced with pseudonyms or codes. Data will not be shared with third parties and will be destroyed after the dissemination of the results. Data destruction will be conducted by reformatting the drive. The study does not involve minors, clinical trials, or any direct physical or psychological risks to participants. No financial compensation will be offered to participants and the research team.

### 2.8. Limitations and Future Research

Despite the rigorous design of this protocol, certain limitations must be acknowledged. First, in Phase 1, the use of convenience sampling may introduce selection bias, as participants who are more accessible or willing to participate may not fully represent the study populations. Second, the reliance on self-report questionnaires could lead to social desirability bias, where respondents may modify their answers to align with social norms. Additionally, the convenience sampling method may lead to an uneven geographical distribution of participants, which could affect the generalizability of the findings across different regions of Italy. However, these limitations are recognized challenges inherent in survey research design [89,90]. Finally, the success of Phase 2 is dependent on participants’ willingness to be re-contacted for interviews, which may lead to a sample biased toward individuals with stronger opinions or greater engagement with the research topic.

These limitations highlight the challenges in conducting large-scale mixed-methods research. To address these issues, future studies could incorporate randomized sampling techniques and longitudinal designs to improve data robustness and generalizability.

## 3. Conclusions

This research protocol presents a new conceptual framework and aims to explore the attitudes of Italian MHNs and NSs toward the use of CMs and STs in psychiatric settings. It also aims to investigate the influence of sociodemographic and psychological factors on these attitudes. The significance of this project lies in addressing critical gaps in the literature, particularly regarding the attitudes and ethical concerns associated with the use of these strategies in Italian mental health nursing practice.

While the actual conclusions will be determined by the study’s outcomes, this research is positioned to make valuable contributions to psychiatric clinical care. Employing both quantitative and qualitative methods, the research will provide a comprehensive understanding of the attitudes and perceptions of Italian MHNs and NSs regarding CMs and STs, offering context-specific insights relevant to Italy while contributing to broader European and international discussions. The analysis of correlations between attitudes, and sociodemographic and psychological factors holds the potential to inform improvements in psychiatric care, enhance safety protocols, and promote the overall well-being of patients and staff. Furthermore, the findings may offer valuable guidance for nursing education, emphasizing the importance of safety management, patient-centered care, and the humanization of mental health services. Finally, the proposed framework could be helpful in conducting future research in this area.

## Figures and Tables

**Table 1:** Structure of the interview.

Subject	Question	Suggestion
Understanding (opening question)	In recent years, there has been a growing interest and use of security technologies in psychiatry. Could you describe what security technologies mean from your perspective?	Could you provide an example of security technologies you are familiar with, perhaps one you have encountered or heard about in your practice?
Perceived benefits	Based on your perspective, what specific benefits do you think security technologies could bring to psychiatric settings?	Can you describe a situation where the use of security technologies could or has directly improved safety or operational efficiency?
Perceived barriers	From your perspective, what barriers might limit the effective deployment of security technologies in psychiatric settings?	Could you discuss any technical or logistical challenges you foresee with the implementation of these technologies?
Ethical consideration	What ethical issues do you foresee or have experienced related to the use of security technologies?	How would you address potential concerns about patient privacy or autonomy when implementing these technologies?
Personal reflections (closing question)	Reflecting on your experiences and perspectives, what do you think about the role of security technologies in modern psychiatric care?	What changes in the use of security technologies would you advocate for?

## Data Availability

No new data were created or analyzed in this study. Data sharing is not applicable to this article.

## Public Involvement Statement

No public involvement in any aspect of this research.

## Guidelines and Standards Statement

This manuscript was drafted against the STROBE guidelines for observational research and the Consolidated Criteria for Reporting Qualitative research (COREQ).
